# PARP Inhibitors and Haematological Malignancies—Friend or Foe?

**DOI:** 10.3390/cancers13215328

**Published:** 2021-10-23

**Authors:** Kathryn A. Skelding, Lisa F. Lincz

**Affiliations:** 1 Cancer Cell Biology Research Group, School of Biomedical Sciences and Pharmacy, College of Health, Medicine and Wellbeing, The University of Newcastle, Callaghan, NSW 2308, Australia; Lisa.Lincz@calvarymater.org.au; 2 Hunter Medical Research Institute, New Lambton Heights, NSW 2305, Australia; 3 Hunter Haematology Research Group, Calvary Mater Newcastle Hospital, Waratah, NSW 2298, Australia

**Keywords:** PARP inhibitors, haematological malignancy, leukaemia, lymphoma, DNA repair

## Abstract

**Simple Summary:**

PARP inhibitors are a class of orally active drugs that kill a range of cancer types by inducing synthetic lethality. The usefulness of PARP inhibitors for the treatment of haematological malignancies has begun to be explored in a variety of both pre-clinical models and human clinical trials. Despite being largely considered safe and well tolerated, secondary haematological malignancies have arisen in patients following treatment with PARP inhibitors, raising concerns about their use. In this review, we discuss the potential benefits and risks for using PARP inhibitors as treatments for haematological malignancies.

**Abstract:**

Since their introduction several years ago, poly (ADP-ribose) polymerase (PARP) inhibitors (PARPi) have become the standard of care for breast and gynaecological cancers with *BRCA* gene mutations. Given that PARPi act by exploiting defective DNA repair mechanisms within tumour cells, they should be ideally suited to combatting haematological malignancies where these pathways are notoriously defective, even though *BRCA* mutations are rare. To date, despite promising results in vitro, few clinical trials in humans for haematological malignancies have been performed, and additional investigation is required. Paradoxically, secondary haematological malignancies have arisen in patients after treatment with PARPi, raising concerns about their potential use as therapies for any blood or bone marrow-related disorders. Here, we provide a comprehensive review of the biological, pre-clinical, and clinical evidence for and against treating individual haematological malignancies with approved and experimental PARPi. We conclude that the promise of effective treatment still exists, but remains limited by the lack of investigation into useful biomarkers unique to these malignancies.

## 1. Introduction

Cells are frequently subjected to DNA damage from a variety of endogenous and exogenous reactive molecules and insults. If this damage is not repaired, the proper functioning of cells can be impaired, which can lead to a variety of deleterious outcomes. Indeed, numerous studies have suggested that cancer progression involves selection of sub-clones with additional mutations that allow them to grow and survive [1]. This clonal evolution is enabled by genomic instability, whereby cells continuously accumulate mutations following DNA damage [2] and is exacerbated by defects in the DNA damage response. Genomic instability is a characteristic of most cancers including haematological malignancies [3].

Several current therapeutic regimens target cancer cells by exploiting defects in the DNA damage response [4]. In this review, we will focus on the potential for poly (ADP-ribose) polymerase (PARP) inhibitors (PARPi) for the treatment of haematological malignancies. Despite the clinical approval of PARPi, which have generally been found to be safe and well tolerated, alarmingly, a small but significant portion of patients with solid tumours treated clinically with PARPi exhibit a variety of haematological toxicities [5]. This highlights how critical it is to better understand the risk of PARPi treatment for different types of cancer before investigating its potential use for haematological malignancies.

## 2. PARP Structure and Function

**Figure 1:**
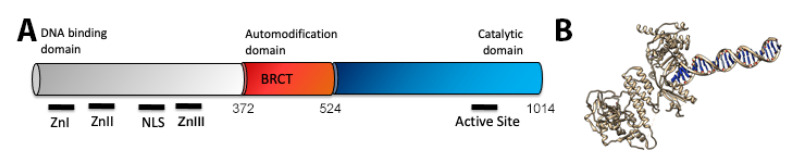
Schematic of PARP-1 structure. (**A**) PARP-1 contains an N-terminal DNA-binding domain (gray), an automodification domain (red) and a C-terminal catalytic domain (blue). The DNA-binding domain encompasses a nuclear localisation signal (NLS) and Cys-Cys-His-Cys zinc finger motifs (Zn) which recognise and bind to damaged DNA. The central automodification domain contains a *BRCA1* carboxy-terminal (BRCT) protein–protein interaction motif. The C-terminal catalytic domain contains a contiguous 50 amino acid sequence, the ‘PARP signature motif’ that comprises the active site. Numbers indicate the amino acid residues for each of the domains. (**B**) Crystal structure of human PARP-1 bound to a DNA double-strand break. Protein Data Bank ID: 4DQY. Generated using Chimera [18].

PARP acts as a DNA damage sensor and caretaker of genomic stability by recruitment of DNA repair machinery to sites of DNA damage [6,7]. PARP is a superfamily of 18 proteins, whose functions and regulation have been extensively reviewed previously [8,9,10,11,12]. The various PARP family members play critical roles in a number of cellular functions, including cell division, regulation of membrane structures, cell viability, cell motility and DNA repair [13]. The main function of the founding member, PARP-1, is poly ADP-ribosylation of various proteins by using nicotinamide adenine dinucleotide (NAD+) as a donor for ADP-ribose [12]. PARP-1 is involved in multiple DNA repair pathways, including the repair of single-strand breaks (SSB), double-strand breaks (DSB) and base excision repair (BER) (reviewed in [10,14,15]). PARP-2 and PARP-3 have also been shown to contribute to SSB repair and BER (reviewed in [9,15]). The first zinc finger in the DNA-binding domain of PARP-1 (ZnI, Figure 1A) is essential for PARP-1 activation by DSB, whereas the second zinc finger (ZnII, Figure 1A) is important for PARP-1 activation by SSB, but not DSB. Upon attachment of the DNA-binding domain of PARP-1 to SSB or DSB (Figure 1B), a conformational change produces an increase in activity that leads to repair of the DNA damage (reviewed in [10]). The central automodification domain is involved in self poly ADP-ribosylation; however, it is not the sole site of ADP-ribosylation. The automodification domain also contains several sites for protein–protein interactions, including domains involved in homo and/or heterodimer formation of PARP-1 with itself or other PARP family members, and a binding site for the breast cancer 1 (*BRCA*-1) protein C-terminus (BRCT) subdomain (Figure 1A) which facilitates the recruitment of X-ray cross-complementing group 1 protein (XRCC1) to act as a scaffold during SSB repair (reviewed in [12,16]). Due to the essential role of PARPs in DNA repair, it is unsurprising that PARPi have emerged over the past decade as promising new anti-cancer therapies for a range of cancer types [17]. 

**Figure 2:**
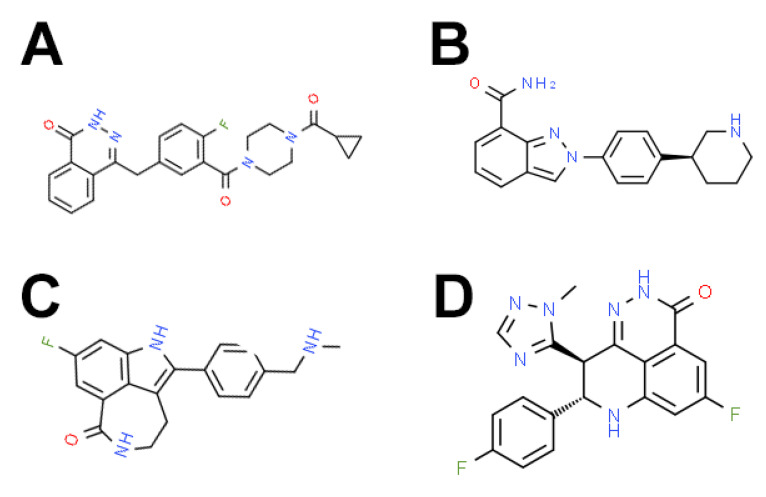
Structure of four PARP inhibitors currently in clinical use. (**A**) Olaparib, (**B**) niraparib, (**C**) rucaparib, and (**D**) talazoparib.

Pharmacological PARPi structurally mimic NAD+, which leads to (i) catalytic inhibition of PARP (i.e., preventing PARylation) and (ii) ‘trapping’ PARP on damaged DNA. Due to this structural mimicry, PARPi generally compete with NAD+ for the catalytic pocket of PARP [19]. Due to the high degree of conservation of the catalytic pocket in the different PARPs, additional interactions are required to ensure selective inhibition [20]. A huge array of potent PARPi have been developed, all of which act in essentially the same manner (reviewed in [17,21,22,23,24,25]). Several of these PARPi have been examined in haematological malignancies, and are summarised in Table 1. In addition to inhibiting the catalytic action of PARP, PARPi can also mediate the trapping of the PARP–DNA complex at replication forks, causing them to stall or collapse [26], resulting in the accumulation of unrepaired SSB, which are eventually converted into DSB. The capacity to form such PARP–DNA complexes varies between different PARPi and positively correlates with their cytotoxic activity. Based on their trapping ability, clinical PARPi can be ranked from most to least potent: talazoparib > niraparib > olaparib = rucaparib > veliparib) (reviewed in [27]). Since 2014, four selective PARPi (Figure 2), olaparib (Lynparza), niraparib (Zejula), rucaparib (Rubraca) and talazoparib (Talzenna), have been approved by the U.S. Food and Drug Administration (FDA) and by the European Medicines Agency (EMA) for a range of cancers (namely, advanced ovarian, metastatic breast, fallopian tube and primary peritoneal cancers). Generally, cancers with high levels of genomic instability and replication stress due to DNA repair deficiencies (especially in homologous recombination repair [HRR]) are particularly responsive to PARPi by a process known as ‘synthetic lethality’ [28,29].

## 3. PARP Inhibitors Use Synthetic Lethality to Kill Cancer Cells with Defects in DNA Repair

**Figure 3:**
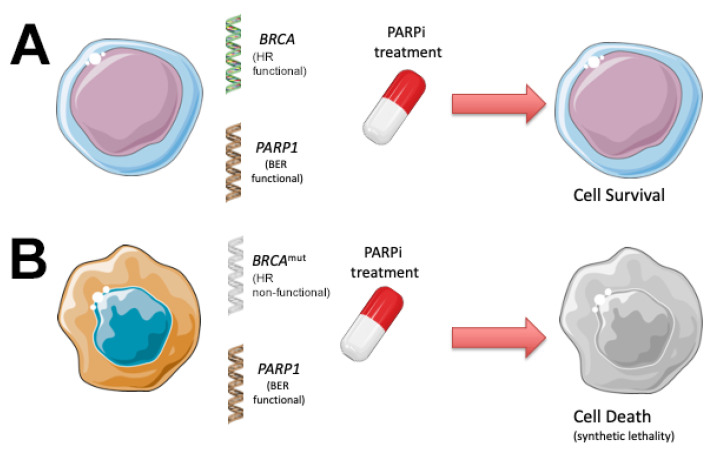
The principle of synthetic lethality—using PARP inhibitors (PARPi) to kill cancer cells with defects in DNA repair. (**A**) Normal cells without *BRCA* mutations have a functioning homologous recombination (HR) repair pathway and a functional base excision repair (BER) pathway. These cells remain alive when treated with PARPi. (**B**) Cancer cells with *BRCA* mutations have a non-functional HR pathway, but a functional BER pathway. When treated with PARPi, these cells are not able to repair DNA damage and subsequently undergo apoptosis.

Currently, most genotype-targeted anti-cancer therapeutics exploit ‘oncogene addiction’, where the cancer cell is dependent on an oncogenic pathway for survival. However, an alternative strategy currently being examined for the treatment of cancer is synthetic lethality [30]. In this strategy, a lethal combination of perturbations in two pathways is exploited as an anti-cancer therapeutic [31]. Mutations in or targeting either pathway alone do not affect cell viability (Figure 3A). However, if one pathway is mutated in cancer cells (such as defects in HRR), the cell becomes overly reliant on other closely related pathways for survival. If one of these related pathways (such as PARP-driven base excision repair [BER]) is pharmacologically inhibited, the tumour cell will die (Figure 3B), whereas normal cells that do not contain the mutation would be spared.

The recent success of PARPi in *BRCA* mutant ovarian cancers is the first clinical example of using synthetic lethality to target tumour suppressor gene loss. *BRCA1* and *BRCA2* are critical components of HRR, which repairs DSB [14]. Cells with mutations in *BRCA1* or *BRCA2* have defective HRR, and thus a predisposition to a range of cancers, including breast and ovarian cancers [32]. *BRCA* mutation status is used to guide treatment decisions for solid tumours (particularly for ovarian and breast cancer) [33,34]; however, whether this is a suitable prognostic in other cancer types where *BRCA* mutations are relatively uncommon, such as in haematological malignancies, remains to be seen.

## 4. PARP Inhibitors as a Potential Treatment for Haematological Malignancies

Despite the promise of PARPi for the treatment of a range of solid tumours with HRR defects, PARPi were not initially evaluated in haematological malignancies due to the rarity of *BRCA1/2* mutations in these cancers [35]. However, emerging evidence shows that the clinical benefits of PARPi are not restricted solely to *BRCA1/2* mutant cancers [36], demonstrating that dysregulation of other key components of HRR or DSB repair pathways may also predict for PARPi sensitivity. Indeed, recent evidence demonstrates that PARPi may also be useful therapeutically for the treatment of a range of haematological malignancies that exhibit defects in HRR and DSB repair.

In addition to the current clinically approved PARPi illustrated above, several other small molecules, such as the phenanthridine-derived compound PJ34, CEP-8983, 6-(5H)-phenanthridinone, AG14361, KU-0058948, the next-generation olaparib AZD-2461, and veliparib (ABT-888) have been developed and examined in a variety of haematological malignancies (Table 1).

### 4.1. Acute Leukaemias

#### 4.1.1. Acute Lymphoblastic Leukaemia

Defects in HRR have been linked to the pathogenesis of acute lymphoblastic leukaemia (ALL). For example, Fanconi Anaemia (FA)-BRCA pathway haploinsufficiency is implicated in the molecular pathogenesis of T-ALL [37], and several polymorphisms in the HRR pathway may affect susceptibility to childhood ALL [38]. This suggests that PARPi may be useful for the treatment of ALL with defective HRR.

Indeed, ALL cells expressing *TCF3-HLF* chimeric mRNAs exhibited defective HRR, specifically by downregulating *MCPH1* expression, thereby leading to decreased *BRCA1/2* expression, and abrogating HRR [39]. As expected, ALL cells expressing *TCG3-HLF* were sensitive to olaparib treatment in vitro, and primary ALL patient blasts were sensitive ex vivo (Table 2) [39,40], suggesting that PARPi may be useful for the treatment of ALL. However, while pre-treatment of ALL patient blasts with olaparib followed by transplantation decreased engraftment of these patient-derived xenografts (PDX) in vivo [40], olaparib monotherapy did not alter survival in *TCF3-HLF* positive HAL-01 and YCUB-2 xenografts in vivo. By contrast, combination of olaparib with temozolomide (TMZ) extended survival in vivo (Table 3) [39]. Taken together, this highlights that PARPi monotherapy is unlikely to be effective clinically, and that the most clinically useful strategy for using PARPi will involve combining with DNA damaging agents and chemotherapeutics.

The PARP-1/2 inhibitor PJ34 has been examined in various models of ALL in vitro (Table 2). While PJ34 treatment did not induce apoptosis in MOLT4 or Jurkat cells in vitro [42,43], it induced apoptosis in human T-cell leukaemia virus type 1 (HTLV-1) transformed and a panel of patient-derived T-ALL cell lines in vitro [41]. This is unsurprising, as HTLV-1 viral proteins have been shown to interact with a variety of DNA repair proteins, including proteins critical for HRR [100,101]. Despite ALL cells largely not being sensitive to treatment with PJ34 alone, it appears that it can sensitise ALL cells to treatment with other agents. For example, combining PJ34 with the histone deacetylase (HDAC) inhibitor vorinostat decreased cell proliferation [42]. By contrast, combination of PJ34 and the NOTCH inhibitor (N-[N[(3,5-difluorophenacetyl)-l-alanyl]-*S*-phenylglycine *t*-butyl ester) (DAPT), or PJ34 and doxorubicin, etoposide, cytarabine, or chlorambucil, did not enhance the cytotoxicity of either drug in Jurkat cells in vitro [43,49]. 

Despite PJ34 demonstrating little effect, other PARPi have shown some promise both in vitro (Table 2) and in vivo (Table 3) for the treatment of ALL. Combination of talazoparib and TMZ in a panel of ALL cell lines in vitro synergistically enhanced cell death [46]. However, these results did not translate to paediatric ALL xenografts in vivo, as excessive toxicity was observed for 7/8 of the xenografts examined, and the remaining xenograft did not demonstrate an objective response [46]. Similarly, veliparib potentiated the effects of TMZ in ALL patient blasts ex vivo and cell lines in vitro [44] but has not been examined in vivo. Furthermore, ALL cells are sensitive to rucaparib in vitro, and combining 5-fluorouracil (5-FU) and rucaparib synergistically enhances cell death in vitro [45]. Whilst rucaparib treatment alone did not increase mouse survival in murine or human PDX transplantation models in vivo, combining with 5-FU did lead to an increase in survival [45]. Similarly, talazoparib monotherapy of *BRCA*/DNA-PK-deficient B-ALL PDX models did not increase survival; however, when combined with doxorubicin and cytarabine, a significant increase in survival was observed in vivo [63]. Taken together, these studies once again highlight that PARPi monotherapy may not be effective for the treatment of ALL patients; however, combination with a variety of cytotoxic chemotherapeutics (such as TMZ and 5-FU) has exhibited some promise and requires further investigation.

#### 4.1.2. Acute Myeloid Leukaemia

Emerging data demonstrate that PARPi are an attractive means of exploiting defects in DNA repair in acute myeloid leukaemias (AML) [14]. For example, AML fusion proteins, such as AML1/ETO and PMLRARα, repress a variety of DNA repair genes and pathways [102,103,104,105,106], and approximately 1/3 of AML cell lines and 11% of primary AML patient samples exhibit microsatellite instability with mono-allelic mutations in two critical HRR components, *CtIP* and *MRE11*, and subsequent downregulation of HRR [60]. Additionally, AML patient samples have been shown to have lower *BRCA1* expression compared to normal bone marrow cells [50]. Taken together, these studies indicate that AML may be particularly amenable to treatment with PARPi.

FA, a haematopoietic disorder that predisposes to myelodysplastic syndrome (MDS) and leukaemia, results from defects in the FA pathway which is also related to HRR [107]. Despite the HRR and the FA pathways being linked, germline mutations of *BRCA* genes, most likely because they are absent from the FA core complex, lead to bone marrow failure less frequently than mutations in other FA genes [108], which offers a potential explanation as to why *BRCA* mutations are less common in AML than in other solid tumours. The investigation of PARPi sensitivity in *BRCA*-proficient AML cells has only recently begun to be explored. Importantly, several common genetic aberrations in AML have been linked to suppression of HRR. AML driven by repressive transcription factors, including AML1-ETO and PML-RARα fusion oncoproteins, and *IDH1/2* mutations, exhibit suppressed HRR [56,109]. *STAG2* (the most frequently mutated subunit of the cohesin complex) mutant AML cells exhibit preferential dependency on multiple members of BER, HRR, mismatch repair and DNA replication machinery, and are exquisitely sensitive to talazoparib in vitro and in vivo [96]. Additionally, *Trp53/Bcor*-mutant acute erythroid leukaemia cells are exquisitely sensitive to talazoparib, alone and in combination with decitabine, in vivo with a reduced spleen size being observed. However, this did not translate into improved survival due to haematopoietic toxicity and sepsis in the combination treated mice [97]. By contrast, FMS-like tyrosine kinase 3 internal tandem duplication (FLT3-ITD) expression in AML is correlated with upregulated RAD51 expression and increased HRR, and inhibiting FLT3-ITD expression results in RAD51 downregulation [110], and inhibited DSB repair [62]. These studies suggest that subtypes of AML may be amenable to treatment with PARPi, and warrants further investigation.

Several PARPi have been examined pre-clinically as potential treatments for AML in a variety of in vitro assays (Table 2) and in vivo xenograft models (Table 3). The PARPi, KU-0058948 and PJ34, induce apoptosis and arrest both established AML cells and primary patient samples in S and G_2_/M phases of the cell cycle [47,48], and delay AML progression in vivo [47]. When PJ34 was combined with the HDAC inhibitors, MS275 or vorinostat, these effects were potentiated [42,48]. By contrast, no potentiation was observed following combination with decitabine, doxorubicin, chlorambucil, cytarabine, or etoposide [42,49], suggesting that not all cytotoxic chemotherapeutics can be combined with PARPi to enhance their effects, and that careful consideration of any potential PARPi and chemotherapeutic combinations is required.

The sensitivity of AML cells to other PARPi remains more controversial. Several studies have demonstrated that clinically relevant doses of olaparib induce apoptosis in AML cells in vitro [50,55,58], including AML1-ETO, PML-RARα and *IDH1/2* mutations, but not MLL or c-KIT mutation, driven AML both in vitro and in vivo [54,56,109]. Additionally, AML patient samples, including those with microsatellite instability, are sensitive to olaparib and talazoparib in vitro [60,63], but PARPi monotherapy did not increase survival in xenograft models in vivo [45,63]. By contrast, other studies have shown that AML cell lines, with the exception of RUNX1-RUNXT1 positive Kasumi-1 cells, are largely resistant to olaparib or veliparib monotherapy [39,52]. These discrepancies can be explained by only particular subtypes of AML being sensitive to PARPi therapy, highlighting the importance of stratifying patients based on their cytogenetic profile when considering treatment with PARPi.

Despite these conflicting findings, a variety of studies have demonstrated that combining olaparib, veliparib, rucaparib and talazoparib with a plethora of chemotherapeutics, including decitabine, TMZ, gemtuzumab ozogamicin (a CD33 antibody linked to calicheamicin), azacytidine, romidepsin, panobinostat, busulfan, melphalan, imatinib, quizartinib, AC220 (FLT3 inhibitor), 5-FU, trichostatin A (TSA), entinostat, avapritinib (c-KIT inhibitor), daunorubicin or irradiation can induce synthetic lethality both in vitro (Table 2) and in vivo (Table 3) [44,45,46,51,52,54,57,58,59,61,62,64,65]. Additionally, combining olaparib with the WEE1 inhibitor AZD1775 significantly increased apoptosis in vitro, decreased tumour burden and improved survival in an AML xenograft model in vivo [55], olaparib and veliparib sensitise AML cells to TRAIL, the recombinant human tumour necrosis factor-related apoptosis inducing ligand [111], and/or combination with vitamin C, synergistically enhanced cell death in vitro [53,58]. As WEE1 inhibitors and TNF-α have been shown to inhibit DNA repair [112,113,114], this enhanced effect of combining PARPi with these agents is not unexpected. Taken together, these studies provide ample evidence for the administration of PARPi with a wide range of anti-cancer drugs, for the treatment of AML. 

Importantly, even resistant AML subtypes can be sensitised to PARPi treatment. For example, suppressing *Hoxa9* expression or inhibiting one of its coregulators, glycogen synthase kinase 3 (GSK3) with LiCl, sensitised MLL-rearranged leukaemia cells (which are traditionally resistant to PARPi) to PARPi in both a primary syngeneic mouse model and a primary human MLL xenotransplantation model [56], and TGFβR1 kinase inhibition (SB431542, galunisertib, TGFβ1-neutralising antibody ID11) re-sensitised AML-bone marrow stromal cell co-cultures to PARPi treatments [64]. By contrast, despite *IDH* mutations being shown to lead to decreased HRR and increase sensitivity to PARPi in AML cells, combination with the *IDH1/2* inhibitors, AGI-5198 and AGI-6780, antagonised the effects of PARPi in vitro [65]. Taken together, these studies highlight the need for further investigation into the usefulness of PARPi as a treatment for AML, so that the most suitable AML subtypes and drug combinations can be identified.

Following on from the emerging pre-clinical evidence demonstrating the potential for PARPi as a treatment for AML, a phase I clinical trial (NCT01139970) in AML examining veliparib + TMZ was conducted (Table 4). This trial demonstrated that combining veliparib with TMZ is well tolerated, with activity in advanced AML [115]. Eight out of 48 (16.6%) patients exhibited a complete remission (CR), with this occurring after 1 cycle in 7/8 patients. Additionally, a further 8/48 (16.6%) patients exhibited a partial response. This shows that this PARPi plus TMZ combination is a promising potential treatment for advanced AML and warrants further clinical investigation. However, it also highlights that additional molecular stratification to identify the subtypes of AML most likely to respond to PARPi treatment are required so that the CR rates in future trials can be increased.

### 4.2. Chronic Leukaemias

#### 4.2.1. Chronic Lymphocytic Leukaemia

Mutations in key DNA DSB repair pathway gene members, including the ataxia telangiectasia mutated (*ATM*) gene, are frequently observed in chronic lymphocytic leukaemia (CLL) [123], and may be associated with worse prognosis, as mouse models of *ATM*-deficient CLL exhibit a significantly earlier disease onset and reduced overall survival (OS) compared to controls [98]. Additionally, CLL samples have reduced *BRCA1* expression compared to non-malignant lymphocytes [72]. This molecular combination results in a ‘BRCAness’ phenotype even in the absence of traditional *BRCA* mutations, suggesting that CLL may be amenable to treatment with PARPi. Indeed, *ATM* mutated CLL cells, including primary CLL patient samples, are sensitive to treatment with olaparib in vitro, and become hypersensitised to bifunctional alkylator bendamustine, fludarabine, 4-hydroperoxycyclophosphamide (4-HC), valproic acid and irradiation [73] (Table 2). Importantly, *ATM*-deficient CLL cells are also sensitive to PARPi in vivo, as olaparib treatment improved spleen volume and survival in a conditional *ATM* knockout mouse model of CLL [98] (Table 3).

CLL cells exhibited varying levels of sensitivity to PARPi in vitro (Table 2). Whilst CLL cells were not sensitive to PJ34 treatment, either as a monotherapy or in combination with DAPT in vitro [43], CLL patient samples stimulated to proliferate with CD40L were sensitive to talazoparib [74], and CEP-8983 displayed single agent cytotoxicity, and a synergistic enhancement when combined with bendamustine in vitro [72]. These findings once again highlight that PARPi are most likely to be beneficial therapeutically when combined with other chemotherapeutics, rather than as a monotherapy.

#### 4.2.2. Chronic Myeloid Leukaemia

Defects in the DNA damage response are a common hallmark of chronic myeloid leukaemia (CML) [124]. For example, a *FANCD2* (plays a crucial role in DSB repair) splice site mutation is associated with CML progression [125], and *BCR-ABL1* oncogenic tyrosine kinases, which are a common oncogenic mutation in CML, induce translational repression and degradation of *BRCA1* proteins [126]. Taken together, this suggests that CML cells may be sensitive to PARPi treatment.

Sensitivity of CML cells to a range of different PARPi have been examined in vitro, with varying levels of promise (Table 2). Imatinib resistant K562 CML cells are sensitive to the PARP inhibitor NU1025 [67]. While PJ34 has no cytotoxic effect on K562 cells [42], it can arrest cells in S and G_2_/M, indicating stalled DNA replication and DNA damage [48]. Additionally, combining PJ34 or KU-0058948 with decitabine did not increase cytotoxicity, but combining with the HDAC inhibitors, MS275 [48] or vorinostat [42], enhanced cell death in vitro. DNA methyltransferase inhibitors (DNMTi), such as decitabine or guadecitabine, enhance talazoparib efficacy in breast and ovarian cancers by augmenting the PARP trapping effects of talazoparib [127]. As olaparib is not as efficient a PARP trapper as talazoparib [27], this lack of an enhanced effect is not surprising. Additionally, combining the PARPi, AG14361, with camptothecin induces growth inhibition and cytotoxicity in K562 cells [66], due to inhibiting BER via the catalytic activity of AG14361.

While CML myeloid crisis-derived cell lines were relatively resistant to treatment with olaparib and veliparib [39], olaparib and a non-NAD-like PARPi, 5F02, can kill CML cells from chronic phase (CML-CP) [68]. In contrast to olaparib, 5F02 exerted limited toxicity against normal Lin-CD34+ cells from healthy donors. Additionally, while combining 5F02 and olaparib induced apoptosis in quiescent DNA-PK-mediated non-homologous end joining (D-NHEJ)-deficient cells, it did not enhance the toxicity against normal cells in vitro. Importantly, this combination exerted synergistic effects in humanised immunodeficient mice bearing *BRCA1*/DNA-PK-deficient primary CML xenografts in vivo [68] (Table 4). Combining olaparib with other chemotherapeutics, such as decitabine [52] or imatinib [68], can induce synthetic lethality in vitro [52]. By contrast, olaparib suppresses cisplatin induced toxicity in CML cells [69] which demonstrates that combining olaparib, or potentially other PARPi, and cisplatin may not be useful for the treatment of CML.

CML-CP cells are modestly sensitive to treatment with talazoparib [70]. Importantly, talazoparib reduced the number of imatininb-refractory CML-CP cells capable of engrafting immunodeficient mice in vivo [70]. Additionally, two paediatric CML patient samples were sensitive to talazoparib treatment, both as a single agent and in combination with the autophagy inhibitor chloroquine, in vitro and in a PDX model in vivo [71].

Taken together these studies indicate that PARPi either alone or in combination with other anti-cancer agents, particularly talazoparib, may be useful for the treatment of CML, including CML in chronic phase, and warrant further investigation.

### 4.3. Lymphomas

Genomic instability and defects in DNA repair pathways are a common hallmark of lymphomas and have been associated with lymphomagenesis [3,128,129,130,131,132,133]. Unsurprisingly, several subtypes of lymphomas have been shown to be sensitive to a variety of PARPi in vitro (Table 2) and in vivo (Table 3).

#### 4.3.1. Non-Hodgkin Lymphoma

Mutations in key DNA repair proteins, including *ATM*, BLM and X-ray repair cross-complementing group 1 (XRCC1), and the nonhomologous end joining (NHEJ)/V(D)J pathway are frequently observed in Non-Hodgkin Lymphoma (NHL), and are associated with susceptibility to the disease [130,134,135,136]. Several additional mutations have been shown to perturb HRR in lymphoma cells. For example, diffuse large B-cell lymphomas (DLBCL) that express LMO2, which inhibits *BRCA1* recruitment to DSB by interacting with 53BP1 during repair, are deficient in HRR [80]. Additionally, *IGH/MYC*-positive Burkitt lymphoma and leukaemia cells exhibit downregulation of *BRCA2* protein [78], suggesting that LMO2 and *IGH/MYC* positive lymphoma cells will be sensitive to PARPi. Indeed, these cells are sensitive to treatment with olaparib in vitro [78,80] and in vivo [80].

A variety of NHL cell lines are sensitive to PJ34, olaparib, talazoparib, and niraparib in vitro, both as single agents [46,57,73,76,77,81] and in combination with doxorubicin, TMZ, ibrutinib, bendamustine, fludarabine, 4-HC, valproic acid and irradiation [46,73,80,81] (Table 2). Further, olaparib enhanced the cytotoxicity of combined gemcitabine, busulfan and melphalan, niraparib synergistically enhanced the effects of decitabine, romidepsin and panobinostat, AG14361 enhanced topotecan-induced cytotoxicity, and 6(5H)-phenanthridinone treatment sensitised RDM4 T-lymphoma cells to irradiation in vitro [57,75,83,84]. The synergistic enhancement observed when combining PARPi and alkylating agents is most likely due to synthetic lethality caused by inhibition of PARP at a time when it is critically required for repairing the SSBs induced by alkylating agents. Olaparib and veliparib treatment also sensitised Raji cells to radiotherapy and ^131^I-tositumomab in vitro [79]. By contrast, the Daudi Burkitt’s Lymphoma mature B-cell line was relatively resistant to treatment with olaparib and veliparib, whereas the Raji cell line was slightly sensitive [39]. This once again highlights the importance of identifying specific subtypes of haematological malignancies that are susceptible to treatment with PARPi, and considering the timing of treatments when combining PARPi with DNA damaging agents.

Importantly, PARPi both alone and in combination with chemotherapeutics has been shown to be effective in a variety of NHL models in vivo (Table 3). While NU1025 treatment alone does not increase survival in an intracerebral syngeneic lymphoma model in vivo, when combined with TMZ, a significant increase in survival was observed [99]. Combination of olaparib with traditional R-CHOP (rituximab, cyclophosphamide, doxorubicin, vincristine, and prednisone) increased survival in 4 DLBCL xenograft models, including a DLBCL PDX model, compared to either treatment alone in vivo [80]. Additionally, olaparib significantly reduced tumour burden and increased survival time in MCL murine xenograft models [73,76,77]. Additionally, talazoparib treatment reduces primary Burkitt lymphoma xenografts in vivo [78], and these effects were synergistically enhanced when combined with cytarabine.

#### 4.3.2. Cutaneous Lymphoma

PARP-1 expression predicts for worse patient survival in cutaneous lymphomas [82,85,137], where chromosomal instability is often observed in cutaneous lymphoma and DSB repair pathways are reduced [138], suggesting that these cancers may be sensitive to treatment with PARPi. Indeed, Sezary syndrome patient samples are sensitive to treatment with the next-generation olaparib, AZD-2461, ex vivo [82]. Additionally, cutaneous T-cell lymphomas are sensitive to talazoparib treatment in vitro and in vivo [85]. Combining talazoparib with the HDAC inhibitor romidepsin synergistically enhances cell death in vitro and in vivo, whereas combination with methotrexate, pralatrexate or bortezomib exhibits antagonistic effects in vitro. Methotrexate inhibits HRR in choriocarcinoma cells [139], so it is surprising that combining methotrexate and PARPi does not lead to a synergistic enhancement of cell death, suggesting that methotrexate may not inhibit HRR in cutaneous lymphoma cells.

#### 4.3.3. Clinical Trials Examining PARPi in Lymphomas

Despite a lack of published pre-clinical evidence, several clinical trials examining veliparib in combination with a variety of chemotherapeutics in lymphoma have been conducted (Table 4). Veliparib has been shown to be well tolerated in a phase 0 study (NCT00387608) in advanced solid tumours (*n* = 8), low-grade lymphoma (*n* = 3) and cutaneous T-cell lymphoma (*n* = 3) [117]. In an additional phase 0 study in NHL (*n* = 3), small-cell lung cancer (*n* = 1), squamous cell carcinoma of the tongue (*n* = 1), melanoma (*n* = 1), cutaneous T-cell lymphoma (*n* = 2) and adenocarcinoma of external ear canal (*n* = 1), veliparib treatment was shown to significantly reduce PAR levels and the ratio of PAR to PARP-1 [116]. However, efficacy was not reported in either of these studies. Additionally, a phase I study (NCT00553189) of veliparib combined with topotecan enrolled 24 patients, including 1 with NHL [118]. Dose-limiting toxicities (DLTs) included grade 4 neutropaenia and thrombocytopaenia, grade 4 neutropaenia lasting longer than 5 days, febrile neutropaenia, and grade 3 or 4 myelosuppression. Effect on NHL disease progression were not reported. A phase I study (NCT00810966) of veliparib in combination with cyclophosphamide in patients with advanced solid tumours (*n* = 33) and refractory lymphoma (*n* = 2) showed that this combination was generally well tolerated, with grade 2 myelosuppression the most common toxicity observed, and 12/35 patients (34%) exhibited grade 3 or 4 lymphopaenia [119]. Additionally, 1 patient with lymphoma exhibited prolonged stable disease, indicating that this treatment regimen may be suitable for the treatment of lymphoma; however, additional examination is required. Furthermore, a Phase 1b clinical trial (NCT01326702) examined veliparib in combination with bendamustine in patients with relapsed/refractory lymphoma (classical Hodgkin Lymphoma [cHL], DLBCL [diffuse large B-cell lymphoma], and FL [follicular lymphoma]), multiple myeloma (MM), with a cohort expansion of bendamustine and veliparib in combination with rituximab in patients with B-cell lymphomas [121]. The combination of veliparib + bendamustine and veliparib + bendamustine + rituximab was generally well tolerated in this trial. Five out of seven (71%) lymphoma patients treated with veliparib + bendamustine and 6/7 (86%) treated with veliparib + bendamustine + rituximab achieved an objective response, and the MM patient achieved a partial response. This trial highlights that these treatment regimens warrant further investigation in a phase II trial.

Olaparib (both a traditional formulation and a new tablet formulation to improve drug loading and bioavailability) was examined in a phase I conventional dose escalation trial (ISRCTN34386131) using a cumulative 3 + 3 design to assess safety and maximum tolerated dose in patients with relapsed CLL (*n* = 9), MCL (*n* = 4) and T-cell prolymphocytic leukaemia (T-PLL) (*n* = 2) [122] (Table 4). Overall, both formulations of olaparib were generally well tolerated with the most common adverse events being anaemia and thrombocytopaenia, and myelosuppression was the most common haematological grade 3–4 toxicity. Median duration of OS in patients who harboured *ATM* or *SF3B1* mutations was 192 days, compared to 89 days for patients without these mutations. This highlights that olaparib may potentially be therapeutically useful for the treatment of lymphoma and warrants further investigation.

### 4.4. Multiple Myeloma

Genomic instability is a characteristic of multiple myeloma (MM) and promotes disease progression and drug resistance [89,140]. Additionally, global HRR defect loss of heterozygosity (HRD-LOH) is associated with impaired outcomes in MM patients. Genome-wide HRD-LOH increases as the disease progresses, and also with higher risk groups [141]. Mutations in key HRR genes (*ATM* and *BRCA2* are the most frequently mutated genes) only account for some of this incidence [141], suggesting that other mechanisms are also responsible. Indeed, RECQ1 helicase, a DNA unwinding enzyme involved in the maintenance of chromosome stability and DSB repair [142], is overexpressed in MM patients and associated with worse OS and event-free survival (EFS) [86], and PARP-1 [89] expression is significantly correlated with poor prognosis of MM patients. Taken together, this suggests that MM may be amendable to treatment with PARPi.

Several PARPi have been shown to induce apoptosis of MM cells pre-clinically (Table 2), including MM that is resistant to existing treatments. For example, RPMI8226/R cells are resistant to several chemotherapeutics, including melphalan. Treatment of RPMI8226/R, but not RPMI8226 cells, with PJ34 inhibited activation of the FA/BRCA pathway, and subsequently sensitised these cells to melphalan treatment in vitro [87]. Additionally, PJ34 treatment sensitised MM cells to dexamethasone in vitro [88]. Decreasing RECQ1 expression in MM cells enhances sensitivity to PJ34 [86], suggesting that inhibitors of RECQ1 helicase may act as sensitisers to PARPi.

Olaparib treatment induces cell death in vitro in a panel of MM cell lines, including bortezomib resistant AMO1 cells, and delays tumour growth in vivo [89]. While veliparib alone failed to induce cell death in a panel of MM cell lines, combining with the cyclin dependent kinase (CDK) inhibitor, dinaciclib, induced synthetic lethality in MM cells, but not normal peripheral B cells [90], and delayed tumour growth and improved survival in vivo. Pharmacological inhibition of the 26S proteasome by bortezomib, induces a ‘BRCAness’ state in MM cells, suppressing HRR, suggesting that this treatment may sensitise them to PARPi [91]. Indeed, while veliparib treatment alone did not induce cell death in vitro, combination with bortezomib resulted in significantly enhanced cell death in vitro and reduced tumour burden and improved survival in vivo. Taken together, these studies demonstrate that MM is especially amenable to PARPi treatment, particularly in combination with 26 proteosome and CDK inhibitors. These findings also highlight the diversity of haematological malignancies and the importance of cytogenetics in determining PARPi sensitivity, as bortezomib did not enhance PARPi effectiveness in cutaneous lymphoma cells.

### 4.5. Myeloproliferative Neoplasms

Myeloproliferative neoplasms (MPNs) represent a heterogeneous group of clonal diseases with a common propensity to progress to acute leukaemia. Primary MPN patient samples exhibit abnormal DNA damage responses, particularly impaired HRR [92], and therapy-related MDS (t-MDS) have a significant downregulation of the *BRCA1–BRCA2–RAD51* axis compared to normal controls [143]. Taken together, this indicates that synthetic lethality may be a promising strategy for treating MPN patients.

Indeed, MPN cells are sensitive to PARPi in vitro (Table 2) and in vivo (Table 3). MPN patient samples, particularly those with impaired RAD51 foci formation, are highly sensitive to treatment with veliparib, olaparib [92] and talazoparib in vitro and in vivo [94]. A new mouse model of MDS where *STAG2* mutations arose as clonal secondary lesions in the background of clonal haematopoiesis driven by ten-eleven translocation methylcytosine dioxygenase 2 (*Tet2*) mutations demonstrated selective depletion of cohesin-mutant cells following treatment with talazoparib in vivo [96]. By contrast, activating *JAK2* mutations correlate with decreased veliparib and olaparib sensitivity in various MPN subsets, particularly myelofibrosis (MF), essential thrombocythaemia (ET), polycythaemia vera (PV), CML, chronic myelomonocytic leukaemia (CMMoL), MDS/MPN-unclassified (MDS/MPN-U). *JAK2V617F* mutations confer significant inter-chromosome HR activity [144], therefore it is not surprising that these activating mutations are correlated with reduced PARPi sensitivity.

Further, several studies have demonstrated that combining PARPi with chemotherapeutics enhances cell death in a variety of models. Combining veliparib and busulfan enhances cell death in primary MF patient samples ex vivo, and in a *JAK2V617F* MPN-AML xenotransplant model in vivo [93], thus demonstrating that inherent resistance to PARPi does not prevent PARPi from sensitising these cells to other anti-cancer drugs. Additionally, combining olaparib and decitabine or olaparib/talazoparib and ruxolitinib and/or hydroxyurea increases cell death compared to either agent alone [94,95].

As pre-clinical evidence indicates that MPNs are amenable to treatment with PARPi, veliparib was examined clinically in combination with topotecan and carboplatin. A Phase I 3 + 3 trial design (NCT03289910) with escalating doses of veliparib combined with topotecan + carboplatin in relapsed or refractory AML, aggressive MPN, or CMMoL demonstrated a response rate was 64% (14/22) for patients with MPN or CMML, and 25% (19/77) for AML with no history of MPN or CMMoL [120]. Mucositis was dose limiting and correlated with high veliparib concentrations. This combination warrants further investigation, particularly in patients with aggressive MPNs, or CMML.

## 5. Treatment of Cancer Patients with PARPi Has Been Associated with Increased Risk of Haematological Toxicities

PARPi have been demonstrated to be clinically effective in a range of solid tumours, with acceptable safety and tolerability in patients. In ovarian cancer patients, a large meta-analysis (*n* = 12 trials; 5347 patients) demonstrated that PARPi significantly improve progression-free survival (PFS) and overall response rate (ORR) compared to placebo and chemotherapies [145], and a second meta-analysis of olaparib, niraparib and rucaparib (*n* = 6 trials; 2270 patients) demonstrated that there are no significant differences in clinical outcomes (OS or PFS) between these three PARPi [146], and together these analyses highlight why PARPi are considered to be the drug of choice in maintenance therapy for platinum-sensitive ovarian cancer. Similar survival benefits have also been observed for metastatic breast cancer [147]. Interestingly, these survival benefits have been identified irrespective of *BRCA* mutation status [148,149]. However, despite these demonstrated survival benefits, concerning haematological toxicities have been observed across a number of studies, which has raised concern as to the suitability of the continued use of these PARPi. 

To help address this controversy, several systematic reviews and meta-analyses have been conducted to evaluate incidence rates and risk ratios for these toxicities in a range of cancer types [145,146,148,150,151,152]. A review of olaparib, veliparib and niraparib phase II and III randomised control trials (RCTs) in ovarian, gastric, non-small-cell lung, and breast cancer and melanoma patients (*n* = 12 trials; 2479 patients) identified that PARPi treatment significantly more than doubled the relative risk (RR) of severe neutropaenia, thrombocytopaenia, and anaemia, when compared to control groups [150]. A further meta-analysis examining the safety of maintenance therapy with olaparib after platinum-based chemotherapy in cancer patients (*n* = 4 trials; 1099 patients with *BRCA* mutated advanced cancers) once again demonstrated that patients treated with maintenance olaparib showed higher risk of all-grade and high-grade anaemia, all-grade neutropaenia and thrombocytopaenia compared to the placebo group [152]. A larger review of phase II and III RCTs in ovarian cancer (*n* = 10 trials; 4553 patients) confirmed these earlier findings and demonstrated that patients treated with PARPi exhibited slight to moderately higher risks of all-grade and high-grade haematological toxicities (including anaemia, leucopaenia, neutropaenia, and thrombocytopaenia with RR ranging from 1.42–3.49) and also marginally increased risks of all-grade gastrointestinal toxicities (including diarrhoea, nausea, vomiting, and constipation with RR ranging from 1.20–1.84) compared to control groups [148]. In addition, a recent large meta-analysis of 29 RCTs (9247 patients) showed that PARPi significantly increases the risk of all-grade anaemia, neutropaenia, and thrombocytopaenia (RR 1.69–2.52) compared to control groups [151]; however, this varied with treatment duration. Taken together, anaemia was the most common haematological toxicity (40–60% of patients reporting anaemia [153]), and all five examined PARPi (veliparib, olaparib, niraparib, rucaparib, talazoparib) were associated with a significantly increased risk of anaemia compared to control groups [151,154]. These meta-analyses overwhelmingly demonstrate that patients treated with PARPi can experience a range of haematological side-effects, and highlight that proper supportive care is essential for these patients.

Other factors must be taken into consideration when assessing the documented risk of haematological toxicities from PARPi treatment. It should be noted that it is not surprising that PARPi increased haematological toxicities in studies where treatment was compared to placebo as a control group, as many chemotherapeutics used to treat a range of cancers induce haematological toxicities. When a more valid control arm of carboplatin and paclitaxel was used for comparison, the risks for many types of haematological toxicities with PARPi monotherapy were no longer significant [150,151], and the toxicities of PARPis as monotherapies appear to be similar to other cytotoxic chemotherapeutic agents. Importantly, the benefits of treatment may outweigh the risk of toxicities: a meta-analysis examining PARPi versus monochemotherapy in patients with *BRCA* mutated HER2-negative metastatic breast cancer (*n* = 2 trials; 733 patients) demonstrated that patients treated with PARPi experienced significantly delayed time to quality of life deterioration, despite a significantly increased risk of anaemia [147]. Similarly, a recent meta-analysis in *BRCA* mutated advanced breast cancer (*n* = 4 trials; 1540 patients) showed that there was no significant difference in the overall adverse events leading to treatment discontinuation when compared to placebo control groups [155], indicating that PARPi are well tolerated and that potential adverse events related to these interventions are generally manageable. However, it is important that these adverse event profiles continue to be closely monitored to ensure that this is the case.

When the adverse event profiles of PARPi plus chemotherapy are compared with chemotherapy alone, the odds of severe anaemia is only slightly higher in combination group than in the chemotherapy alone (odds ratio [OR 1.55, 95%CI 1.17–2.05]) [154]. By contrast, the odds of severe nausea was halved (OR 0.51, 95% CI 0.26–1.00) in this combinatorial group. Taken together, these findings highlight that different combinations are likely to produce unique adverse event profiles, and clinicians will need to carefully consider the needs of the patient when making treatment decisions.

Interestingly, different PARPi exhibited varying toxicities. Niraparib exhibited a significantly higher risk of suffering high grade thrombocytopaenia [146,148,150] and neutropaenia [146], whereas leucopenia was not observed in patients treated with niraparib or rucaparib [148]. Olaparib was associated with the highest risk of neutropaenia [150] and total grade 3 or greater adverse events [145,152]. Overall, niraparib and talazoparib have more prominent haematological adverse event profiles, rucaparib was associated with major abdominal pain events, olaparib with diarrhoea, and niraparib was also associated with an increased risk of cardiac events [156,157]. Haematological adverse events are linked to the PARPi potency of PARP-1 trapping [158], which also most likely contributes to their effectiveness for the treatment of haematological malignancies. These haematological adverse events are most frequent in the initial months of treatment and decline over time [159]. Therefore, early monitoring of patients treated with PARPi are advisable so that dose reduction or treatment discontinuation can be advised if required.

Despite the evidence that PARPi induce haematological toxicities for the treatment of solid tumours, they should not be immediately ruled out for the treatment of haematological malignancies due to these toxicities. Current treatments for haematological malignancies induce a comparable level of haematological toxicities as has been seen with PARPi. For example, 45% of AML patients treated with azacytidine + Venetoclax experienced grade 3 or higher thrombocytopaenia, and 42% suffered febrile neutropaenia [160], while cytarabine treatment for CNS lymphoma caused grade 3 or 4 haematological toxicities of thrombocytopaenia, anaemia, and neutropaenia in 79%, 71% and 57% of patients, respectively [161]. These incidences are comparable or higher than what are observed following PARPi treatment in solid cancers, which include anaemia (30–60% of patients), neutropaenia (~20%), or thrombocytopaenia (15%) [153]. However, the incidence of PARPi-induced hematological toxicities in patients with reduced bone marrow capacity due to haematological disease has not been widely examined, and may be comparable with existing chemotherapeutics. This highlights that haematological toxicity does not preclude the use of current drugs for the treatment of haematological or solid tumours if the survival benefit outweighs the risk of toxicity.

Although PARPi have been developed to promote synthetic lethality in tumours that already harbour defects in DNA repair, it stands to reason that any drug that interferes with essential DNA repair processes risks the accumulation of DNA mutations that may lead to carcinogenesis in an otherwise healthy cell. Concerningly, the development of MDS and AML has been observed in patients following treatment with PARPi [159,162,163]. However, the incidence of AML/MDS development/death is rare; 0.3% of patients treated with talazoparib developed MDS/AML [156], the death of 1/298 (0.3%) of patients in a phase II trial was attributed to olaparib-related MDS [164], 1% of patients developed MDS/AML following olaparib treatment in the SOLO1 trial [165], 0.8% of patients treated with rucaparib in the ARIEL3 study developed MDS/MDL [166,167], and the overall risk of AML/MDS is 0.9% of all patients treated with niraparib [165]. A meta-analysis with a total of 3 phase III trials in patients with recurrent ovarian cancer, failed to show any statistically significant increased risk of secondary haematological malignancies with the use of PARPi compared to a placebo control arm (Mantel-Haenzel [MH] risk ratio 1.14, 95% CI 0.42–3.08) [168]. A second meta-analysis examining three phase III RCTs (1401 patients) of olaparib, niraparib or rucaparib found that while there was an increase in the risk of all grades of haematological toxicities, no significant increase in the risk of secondary haematological malignancies were observed with PARPi treatment, when compared to placebo control [169]. By contrast, a larger meta-analysis and retrospective pharmacovigilance study examining 18 placebo and 10 non-placebo randomised control trials (RCTs) and 211 cases in the World Health Organisation (WHO) pharmacovigilance database (VigiBase) demonstrated that PARPi increase the risk of MDS and AML when compared to placebo treatment (Peto OR 2.63, 95% CI 1.13–6.14). However, when compared to non-placebo controls (i.e., chemotherapeutic controls), the Peto OR reduced to 1.33 (95% CI 0.28–6.38). The median latency period for the development of these haematological abnormalities was 20.3 months (18.4–26.6 months). The majority of reported patients (MDS: 76%; AML: 73%) were treated with olaparib [5]. In addition, the emergence of clonal haematopoiesis was associated with PARPi in 16/18 (78%) of ovarian cancer patients who received PARPi maintenance therapy (median, range = 11.2, 0.4–45.8 months), compared to 7/18 patients (39%) who did not (*p* = 0.018). Sequencing revealed an overrepresentation of mutations in the DDR pathway (*p* = 0.002) in the PARPi treated patents, with mutations that emerged or expanded during the course of therapy [5]. While this is a concerning finding, it should be noted that MDS/AML risks are significantly elevated after standard chemotherapy for 22 of 23 solid cancers [170], thus suggesting that the increased risk of AML/MDS is not just restricted to PARPi treatment. However, the median latency period since first exposure to a PARPi was 17.8 months (8.4–29.2) [5], and for other chemotherapeutics is typically 3–5 years [171], a possible reflection of the heavily pre-treated cohorts enrolled in these early phase trials. As many of the PARPi trial patients had received prior chemotherapy, these adverse events and shorter median latency times could be associated with previous lines of chemotherapy. With that being said, clinicians should assess the potential risk of secondary haematological malignancies when evaluating the risk-benefit ratio for using PARPi.

The majority of the patients presenting with haematological toxicity possessed germline *BRCA* mutations [172], suggesting that this may be contributing to the development of these haematological abnormalities. PARP inhibition may allow some *BRCA* mutated cells to survive with non-lethal mutations that eventually lead to leukaemia [173]. Therefore, if additional markers of PARPi sensitivity can be identified, this toxicity may be able to be eliminated. However, this remains to be confirmed, and highlights that this significant risk of developing MDS or AML warrants further investigation. 

## 6. Conclusions

The evidence ‘for’ far outweighs the evidence ‘against’ the use of PARPi for the treatment of a range of cancers; however, the right patients need to be targeted for optimal treatment with minimal haematological toxicity. In general, PARPi performed better when used in combination with other drugs than when used alone for the treatment of a wide variety of haematological malignancies, with only two studies reporting an antagonistic effect whereby olaparib blocked cisplatin-induced cell death in CML and *IDH1/2* mutation inhibitors blocked olaparib and talazoparib in AML cell lines. Despite the inclusion of patient samples in 20 of the listed publications, only a few studies attempted to identify molecular markers of PARPi susceptibility, the majority of which investigated biomarkers for olaparib. Notable findings in AML included increased sensitivity in cells harbouring AML1-ETO alone compared to AML1-ETO + C-KIT mutation [54], and in cells with FLT3-ITD compared to FLT3-WT [62]. Cells with *IDH1/2* mutation were also susceptible to olaparib, but this was antagonised when combined with *IDH1/2* inhibitors [65]. In CLL, *ATM* deficiency correlated with olaparib sensitivity [73], and in MPN, *JAK2V617F*, CALR (del52), MPL (W515L) identified patient samples most likely to benefit from combined therapy with ruxolitinib and hydroxyurea [94]. There is clearly scope for identification of more PARPi and disease-specific biomarkers. However, this limited series suggests the existence of complex pathways that warrant further investigation to form a comprehensive understanding of how different PARPi function in the setting of the particular genetic alterations that characterise various haematological malignancies.

Of the 27 in vivo investigations of PARPi as monotherapy for haematological malignancies listed in Table 3, 12 showed no effect. However, 22/23 (95.7%) combination studies showed greatly increased survival when PARPi was used with other common chemotherapeutics, with only one negative study reporting excessive toxicity when talazoparib was combined with TMZ [46]. In contrast to the wider variety of PARPi tested in vitro, pre-clinical in vivo studies were limited to talazoparib (*n* = 14), olaparib (*n* = 11), rucaparib (*n* = 8), and veliparib (*n* = 3), with only 3 reports using more experimental PARPi; PJ34, SF02 or NU1025. Although the majority of studies used patient-derived samples, in most instances, there was limited molecular information provided or stratification employed. This is most likely due to the limited understanding of predictive biomarkers for the use of PARPi in haematological malignancies and highlights the need for a better understanding of HRR and PARPi sensitivity in these cell types.

Despite what appeared to be promising pre-clinical results across all the PARPi tested in vivo, only veliparib (*n* = 6) and olaparib (*n* = 1) have published results (2009–2018) on trials in humans with haematological malignancies (Table 4). These early phase trials mainly included AML, MPN, and lymphoma patients, and 4/7 used combinations of PARPi with other chemotherapeutics. PARPi were generally well tolerated and showed good oral bioavailability, and where evaluable, response rates ranged from 16–25% for AML (*n* = 48 and *n* = 77). Exploratory studies indicated that clinical response was best predicted by higher treatment induced histone H_2_AX phosphorylation [115,120]. Further investigation into the use of these drugs for haematological disorders with inherent DNA repair defects and how to predict these responders is an emerging field of interest that warrants further clinical investigation.

## Figures and Tables

**Table 1:** PARP inhibitors investigated in haematological malignancies.

PARP Inhibitor	Other Names	IUPAC/Chemical Name	Target	Haematological Malignancy
Olaparib	AZD-2281, KU-59436	4-[[3-[4-(cyclopropanecarbonyl)piperazine-1-carbonyl]-4-fluorophenyl]methyl]-2H-phthalazin-1-one	PARP-1/2/3	ALL, AML, CLL, CML, T-cell lymphoma, MM, MPN, NHL
Rucaparib	AG-14699, PF-01367338	8-gluoro-2-(4-((methylamino)methyl)phenyl)-4,5-dihydro-1H-azepino[5,4,3-cd]indol-6(3H)-one phosphate	PARP-1/2/3	ALL, AML, ALL
Niraparib	MK4827	(S)-2-(4-(piperidin-3-yl)phenyl)-2H-indazole-7-carboxamide	PARP-1/2	AML, NHL
Talazoparib	BMN-673, MDV-3800	((8S,9R)-5-fluoro-8-(4-fluorophenyl)-9-(1-methyl-1H-1,2,4-triazol-5-yl)-8,9-dihydro-2H-pyrido[4,3,2-de]phthalazine-3(7H)-one	PARP-1/2	ALL, AML, CLL, CML, NHL, T-cell lymphoma, MPN
Veliparib	ABT-888	2-[(2R)-2-methyl-2-pyrrolidinyl]-1H-benzimidazole-7-carboxamide	PARP-1/2	ALL, AML, CML, MM, MPN, NHL, cutaneous T-cell lymphoma, refractory lymphoma, DLBCL, cHL, FL, tFL
CEP-8983	CK-102	11-methoxy-4,5,6,7-tetrahydro-1H-cyclopenta[a]pyrrolo[3,4-c]carboazole-1,3(2H)-dione	PARP-1/2	CLL
PJ34	-	N-(6-oxo-5,6-dihydrophenanthridin-2-yl)-N,N-dimethyl acetamide	PARP-1/2 Phenanthridine PARS inhibitor, tankyrase-1/2	ALL, AML, CLL, CML, MM
5F02	-	1-(2-(cyclododecyloxy)-2-oxoethyl)-1-methylpiperidin-1-ium iodide	PARP-1 (non-NAD-like)	CML
AG14361	-	1-(4-((dimethylamino)methyl)phenyl)-8,9-dihydro-2,7,9a-triazabenzo[cd]azulen-6(7H)-one	PARP-1	CML
6-(5H)-phenanthridinone	NSC 11021, NSC 40943, NSC 61083, PHEN	5H-phenanthridin-6-one	PARP-1	T-cell lymphoma
KU-0058948	Homopiperazine analogue, 14	4-[[4-fluoro-3-[(hexahydro-1H-1,4-diazepin-1-yl)carbonyl]phenyl]methyl]-1(2H)-phthalazinone	PARP	AML, CML
NU1025	NSC 696807	8-Hydroxy-2-methyl-4(3H)-quinazolinone	PARP	CML, murine lymphoma
AZD-2461	1174043-16-3	4-(4-fluoro-3-(4-methoxypiperidine-1-carbonyl)benzyl)phthalazine-1(2H)-one	PARP	T-cell lymphoma

ALL, acute lymphoblastic leukaemia; AML, acute myeloid leukaemia; cHL, classical Hodgkin lymphoma; CLL, chronic lymphoblastic leukaemia; CML, chronic myeloid leukaemia; DLBCL, diffuse large B-cell lymphoma; FL, follicular lymphoma; tFL, transformed follicular lymphoma; MM, multiple myeloma; MPN, myeloproliferative neoplasms; NHL, non-Hodgkin lymphoma.

**Table 2:** Summary of pre-clinical in vitro investigations of PARPi for haematological disorders. + induced apoptosis or inhibited cell proliferation; - no effect; N/A combination not examined; ++ combination synergistically increased effects; ± combination did not increase effect.

Cancer	Cell Lines	Drugs		Effect	Mono	Combo	Refs
		PARPi	Chemotherapeutic				
Acute leukaemias							
ALL	MT-4, MT-2, C8166, C91PL, MT-1, ATL-T, ED-40515(-), ALT-25, ATL-43T, KOB, ATL-55T	PJ34	-	Induced apoptosis and cell cycle arrest	+	N/A	[41]
	MOLT4	PJ34	Vorinostat	PJ34 alone had no effect. Combining PJ34 and vorinostat decreased proliferation	-	++	[42]
	Jurkat	PJ34	DAPT	No effect either as monotherapy or in combination with DAPT	-	-	[43]
	Patient-derived blasts, Jurkat, MOLT-4, MOLT-3, CCRF-CEM	Olaparib	-	Induced apoptosis	+	N/A	[40]
	MOLT3, Jurkat, NALM-6, Reh, KOPN60, KOPN36, YCUB-2, HAL-01, KOCL-58, RS4;11, KOPB-26, KOCL-33	Olaparib, veliparib	-	Induced apoptosis	+	N/A	[39]
	Patient-derived blasts, Jurkat, MOLT-4, HSB2	Veliparib	TMZ	Veliparib monotherapy inhibited leukaemia cell growth and potentiated the effects of TMZ	+	++	[44]
	RPMI-8402	Rucaparib	5-FU	Sensitive to rucaparib alone, and cell death increased when combined with 5-FU	+	++	[45]
	NALM-6, COG-LL-317, RS4;11, MOLT4, CCRF-CEM	Talazoparib	TMZ	Combination enhanced cell death	+	++	[46]
AML	THP-1, Kasumi-1	PJ34	-	PJ34 suppressed proliferation and induced apoptosis	+	N/A	[47]
	HL60, K562, NB4, U937, Kasumi, OC-1, Raji, KG-1, ME-1, P39, Mutz-3, OCI-AML3, Primary patient samples	KU-0058948, PJ34	Decitabine, MS275	Decitabine did not potentiate the effects of PARPi. MS275 enhanced the effects of PARPi in all PARPi sensitive cells	+	Decitabine: - MS275: ++	[48]
	U937, HL60	PJ34	Vorinostat	PJ34 alone had no effect. Combining PJ34 and vorinostat decreased proliferation in HL60 cells	-	++	[42]
	HL60	PJ34	Doxorubicin, etoposide, cytarabine, or chlorambucil	PJ34 alone decreased cell survival. Combination did not significantly enhance cytotoxicity	+	±	[49]
	HL-60, U937, NB4, OCI-AML2, OCI-AML3, Patient samples	Olaparib	-	Olaparib induced apoptosis	+	N/A	[50]
	HL60	Olaparib	Gemtuzumab ozogamicin	Olaparib treatment as a monotherapy did not induce apoptosis. Combination with gemtuzumab ozogamicin synergistically enhanced cell death	-	++	[51]
	KG1a, MV-4-11, PL21, HL60	Olaparib	Decitabine	Olaparib alone had minimal effect. Combining decitabine and olaparib induced apoptosis	-	++	[52]
	HL60, MOLM13, THP1, KG1	Olaparib	Vitamin c	Vitamin C treatment enhanced sensitivity to olaparib	+	++	[53]
	Patient samples	Olaparib	Avapritinib	Olaparib alone has little effect in patient samples harbouring AML1-ETO and a c-KIT mutation, but induced apoptosis in AML1-ETO AML cells. Combining olaparib and avapritinib increased cell death	AML1-ETO: + AML1-ETO + c-KIT^MUT^: -	++	[54]
	MOLM13, MV4-11, REH, OCI-AML3	Olaparib	AZD1775	Olaparib alone induced cell death. Combining olaparib and AZD1775 significantly enhanced cell death	+	++	[55]
	THP-1, Kasumi-1, AML patient samples	Olaparib, veliparib	-	AML1-ETO and PML-RARα-driven AML were sensitive to PARPi. MLL-driven AML was not	AML1-ETO, PML-RARα: + MLL: -	N/A	[56]
	KG-1, ML-1, Kasumi-1, THP-1, U-937, CMK, NB4, HL60, ML1	Olaparib, veliparib	-	Not sensitive, with the exception of Kasumi-1 cells	-	N/A	[39]
	OCI-AML2	Rucaparib	5-FU	Sensitive to rucaparib alone, and combination with 5-FU enhanced cell death	+	++	[45]
	AML patient blasts; U937, HEL, THP-1, KG-1, HL60	Veliparib	TMZ	Veliparib monotherapy inhibited leukaemia cell growth and potentiated TMZ	+	++	[44]
	KBM3/Bu250, MOLM14	Niraparib	Romidepsin, Panobinostat, decitabine, busulfan, melphalan	Combination synergistically inhibited cell proliferation	+	++	[57]
	NB4 ATO-sensitive and resistant clones	Olaparib, talazoparib, veliparib, niraparib, rucaparib	Decitabine, azacytidine, ascorbate	All clones were sensitive to olaparib, niraparib and talazoparib, but not to rucaparib and veliparib. Combining PARPi with hypomethylating agents induced synergistic growth inhibitory effects in both ATO-sensitive and resistant clones. Combining ascorbate with niraparib and talazoparib synergistically enhanced effects	Ola: + Nir: + Tal: + Ruc: - Vel: -	++	[58]
	Kasumi-1, MV-4-11, MOLM13, MOLM14	Veliparib, talazoparib	Azacytidine	Low doses of azacytidine and PARPi increased cell death	+	++	[59]
	Patient samples, KG-1, MOLM-13, NB4, OCI-AML2, OCI-AML, P39	Talazoparib	-	Cells with microsatellite instability were hypersensitive to PARPi	+	N/A	[60]
	Kasumi-1	Talazoparib	TMZ	Combination enhanced cell death	+	++	[46]
	K563, HL60, Patient samples	Talazoparib	TSA, Entinostat	Talazoparib alone did not induce cell death. Combining with TSA or entinostat significantly increased apoptosis	-	++	[61]
	MV-4-11, HL60, REH, Patient samples	Olaparib, talazoparib	AC220	REH cells were highly sensitive to talazoparib. FLT3-ITD cells were more sensitive to olaparib than FLT3-WT cells. Combining with AC220 significantly increased apoptosis in all cells	+	++	[62]
	Patient samples	Olaparib, talazoparib	-	PARPi-induced apoptosis	+	N/A	[63]
	Patient samples, K562, Kasumi-1	Olaparib, talazoparib	SB431542	Cells were sensitive to olaparib and talazoparib. When co-cultured with bone marrow stromal cells, AML cell sensitivity to PARPi decreased. This was restored by combined treatment with SB435142	+ Co-culture: -	++	[64]
	Patient samples (*IDH1/2*^MUT^ and *IDH1/2*^WT^)	Olaparib, talazoparib	Daunorubicin, irradiation, AGI-5198, AGI-6780	*IDH1/2* mutant cells were sensitive to PARPi, and this was enhanced via combination with daunorubicin or irradiation. *IDH1/2* inhibitors antagonised this effect	+	DNR: ++ Irrad: ++ IDH inhibitors: --	[65]
Chronic leukaemia							
CML	K562	PJ34	Vorinostat	PJ34 had no effect. Combining PJ34 and vorinostat decreased proliferation	-	++	[42]
	K562	KU-0058948, PJ34	Decitabine, MS275	PARPi-induced cell cycle arrest and apoptosis. Addition of decitabine failed to increase cytotoxicity of PARPi. Combination with MS275 potentiated the cytotoxic effect of PARPi	+	Dec: - MS275: ++	[48]
	K562	AG14361	Camptothecin	AG14361 enhanced the growth inhibitory and cytotoxic effects of camptothecin	N/A	++	[66]
	K562, K562 imatinib resistant, Mo7e-P210 IMR2	NU1025	L67	Cells were sensitive to NU1025, and combining with L67 significantly increased cell death, even in imatinib resistant cell lines	+	++	[67]
	Patient samples	5F02, olaparib, talazoparib	-	PARPi-induced apoptosis as single agents. Combining 5F02 and olaparib induced apoptosis in quiescent CML cells	+	N/A	[68]
	K562	Olaparib	Decitabine	Olaparib alone had minimal effect. Combining decitabine and olaparib induced apoptosis	-	++	[52]
	BV173, K562, AR230, MEG-01	Olaparib, veliparib	-	Induced apoptosis in all cell lines except K562	+	N/A	[39]
	K562, MEG01	Olaparib	Cisplatin	Olaparib blocked cisplatin-induced cell death	+	--	[69]
	Patient samples grown on a HS-5 stromal cell monolayer	Talazoparib	Imatinib	Talazoparib inhibited the clonogenic potential of imatinib-refractory CML cells	+	++	[70]
	Paediatric patient samples	Talazoparib	Chloroquine	Talazoparib monotherapy induced cytotoxicity. Combination synergistically enhanced cell death	+	++	[71]
Lymphomas							
CLL	CLL, 697	PJ34	DAPT	No effect either as monotherapy or in combination with DAPT	-	-	[43]
	Patient samples	CEP-8983	Bendamustine	CEP-8983 induced cytotoxicity as a single agent. Synergistically enhanced cell death when combined with bendamustine	+	++	[72]
	Patient samples, PGA (parental and *ATM* knockdown)	Olaparib	4HC, fludarabine, valproic acid, bendamustine and irradiation	*ATM*-deficient cells were sensitive to olaparib. Olaparib sensitised cells to treatment with 4HC, fludarabine, valproic acid, bendamustine and irradiation	+	++	[73]
	Patient samples	Talazoparib	-	Induced cell death	+	N/A	[74]
NHL	Granta-519, Jeko1, JVM2, Z138C	AG14361	Topotecan	AG14361 potentiated topotecan cytotoxicity independently of *TP53* or either *ATM* or *BRCA2* knockdown/inhibition	N/A	++	[75]
	Granta-519, HBL-2, JVM-2, MAVER-1, Z138, C35ABR, L3	Olaparib, PJ34	-	Induced cell death in *ATM*-deficient cells	+	N/A	[76]
	C35ABR, UPN1, UPN2, Granta-519, HBL-2, JVM-2, Z138, L3	Olaparib	-	Induced cell death in *ATM*-deficient cells	+	N/A	[77]
	Raji, Daudi	Olaparib, veliparib	-	Induced apoptosis	+	N/A	[39]
	Mutu, Raji, DG75, and patient samples	Olaparib, talazoparib	-	Decreased cell survival	+	N/A	[78]
	Raji	Olaparib, veliparib	External source caesium-based radiation and ^131^I-tositumomab	PARPi sensitised cells to irradiation	+	++	[79]
	OCI-LY1, OCI-LY8, SUDHL-6, G452, VAL, DOHH2, U2932, OCI-LY19, HCC1187	Olaparib	Doxorubicin	Olaparib alone decreased proliferation and colony formation in LMO2 expressing cells. Combination with doxorubicin synergistically enhanced these effects.	+	++	[80]
	Granta-519, JVM-2	Olaparib	4HC, fludarabine, valproic acid, bendamustine and irradiation	*ATM*-deficient cells were more sensitive to olaparib that *ATM* competent cells. Olaparib sensitised cells to treatment with 4HC, fludarabine, valproic acid, bendamustine and irradiation	+	++	[73]
	Granta-519, Z-138	Olaparib	Ibrutinib	Olaparib exhibited cytotoxicity as a single agent. Combining olaparib and ibrutinib synergistically enhanced cell death	+	++	[81]
	Toledo	Niraparib	Romidepsin, Panobinostat, decitabine, busulfan, melphalan	Combination synergistically inhibited cell proliferation	+	++	[57]
	Karpas-299, RAMOS-RA1	Talazoparib	TMZ	Enhanced cell death with combination	+	++	[46]
T-cell lymphoma	Sezary Syndrome Patient samples	AZD-2461	-	Induced cell death	+	N/A	[82]
	RDM4	6(5H)-phenanthridinone	^60^Co panoramic γ-radiation	PARPi sensitised cells to irradiation	+	++	[83]
	J45.01, Toledo, T-cell lymphoma Patient samples	Olaparib	Gemcitabine, busulfan, melphalan	Olaparib enhanced the cytotoxicity of combined gemcitabine, busulfan, and melphalan	+	++	[84]
	MBL2, HUT78, EL4,	Talazoparib	Bexarotene, vorinostat, romidepsin, methotrexate, pralatrexate, bortezomib	Talazoparib arrested the cell cycle at G_2_/M. Combining talazoparib with romidepsin or bexarotene synergistically enhanced cell death. Combining talazoparib with vorinostat, methotrexate, pralatrexate or bortezomib exhibited antagonistic effects.	+	Rom: ++ Bex: ++ Vor: -- Meth: -- Pra: -- Bort: --	[85]
Multiple Myeloma							
	XG7, XG19	PJ34	-	PJ34 treatment slightly decreased cell viability	+	N/A	[86]
	RPMI8226/R, RPMI8226	PJ34	Melphalan	PJ34 enhanced the cytotoxicity of melphalan in RPMI8226/R cells but not RPMI8226 cells	+	++	[87]
	RPMI-8226, MM1.S	PJ34	Dexamethasone	PJ34 enhanced the cytotoxicity of dexamethasone	+	++	[88]
	CAPAN1, H929, OPM2, U266m R8226, INA6, KMS26, M12BM, KMS11, bortezomib resistant AMO1 cells	Olaparib	-	Majority of cell lines (except U266, KMS11, OPM2) were sensitive to olaparib	+	N/A	[89]
	NCI-H929, RPMI-8226, MM.1S, normal human peripheral CD19^+^ B cells	Veliparib	Dinaciclib	Veliparib alone did not induce cell death. Combining with dinaciclib induced synthetic lethality in MM cells, but not normal peripheral B cells	-	++	[90]
	NCI-H929, RPMI-8226, KMS11, MM1S, OPM2	Veliparib	Bortezomib	Veliparib alone had limited effect, but combining with bortezomib significantly increased cell death	-	++	[91]
Myeloproliferative Neoplasms							
	*BCR/ABL*+ CML, ET, PV, primary and secondary MF and mixed MDS/MPN patient samples	Veliparib, olaparib	-	MPN patient samples with impaired RAD51 foci were particularly sensitive to treatment with PARPi	+	N/A	[92]
	MF patient samples	Veliparib	Busulfan	MF patient samples were sensitive to PARPi treatment alone. Combining with busulfan significantly enhanced these effects	+	++	[93]
	*JAK2V617F*, CALR (del52), MPL (W515L) patient samples	Olaparib, talazoparib	Ruxolitinib, hydroxyurea	MPN samples were sensitive to PARPi treatment. Combining with ruxolitinib and hydroxyurea significantly enhanced sensitivity of MPN cells to PARPi	+	++	[94]
	Patient samples	Olaparib	Decitabine	Olaparib alone induced cytotoxicity and was enhanced by combining with decitabine	+	++	[95]

ALL, acute lymphoblastic leukaemia; AML, acute myeloid leukaemia; *ATM*, ataxia telangiectasia mutated; ATO, arsenic trioxide; CLL, chronic lymphoblastic leukaemia; CML, chronic myeloid leukaemia; ET, essential thrombocythaemia; 5-FU, 5-fluorouracil; MCL, mantle-cell lymphoma; MDS, myelodysplastic syndrome; MF, myelofibrosis; MM, multiple myeloma; MPN, myeloproliferative neoplasms; NHL, non-Hodgkin lymphoma; PARPi, PARP inhibitor; PV, polycythaemia vera; TMZ, temozolomide; TSA, trichostatin A.

**Table 3:** Summary of pre-clinical in vivo investigations of PARPi for haematological disorders. + induced apoptosis or inhibited cell proliferation; - no effect; N/A combination not examined; ++ combination synergistically increased effects.

Cancer	Model	Treatment Regimen		Effect	Mono	Combo	Refs
		PARPi	Chemotherapeutic				
Acute Leukaemias							
ALL	NSG mice, patient-derived T-ALL samples pre-treated with olaparib i.v.	Pre-treatment with 5µM olaparib for 48 h prior to transplantation	-	Decreased engraftment	+	N/A	[40]
	Female NSG mice, HAL-01 or YCUB-2 cells i.v.	Olaparib (100 mg/kg) orally 5 times per week	TMZ (25 mg/kg) orally 5 times per week	Olaparib and TMZ alone no effect. Combination significantly increased survival	-	++	[39]
	(1) Female and male C57BL6/Ly5.1 mice transplanted with spleen cells from ALL ENU treated mice (2) Female and male NSG mice, patient-derived T-ALL sample i.v.	(1) Rucaparib (1 mg/kg) i.p. for 5 days (2) Rucaparib (1.3 mg/kg) i.p. for 5 days	(1) 5-FU (150 mg/kg) i.p. on day 2 (2) 5-FU (75 mg/kg) i.p. on day 2	Rucaparib alone had no effect in either model. Combination with 5-FU increased survival in both models	-	++	[45]
	Female NOD.CB17-Prkdc^scid^/J mice, 8 patient-derived ALL samples i.v.	Talazoparib (0.25 mg/kg) twice daily	TMZ (12 mg/kg) daily × 5	No objective response, and excessive toxicity for 7/8 xenografts	-	-	[46]
	NSG mice, patient-derived ALL cells i.v.	Talazoparib (0.33 mg/kg) orally for 7 days	Imatinib (100 mg/kg) orally twice daily	Talazoparib alone increased survival. Combination with imatinib further increased survival	+	++	[63]
AML	Male C57Bl/6J mice, murine C1498 cells i.v.	PJ34 (10 mg/kg) i.p. daily for four weeks	-	PJ34 increased survival and delayed tumour progression	+	N/A	[47]
	Female and male NSG mice, (1) Kasumi cells i.f., (2) THP-1 i.v., (3) patient-derived MLL-AML samples i.f.	(1, 2) Olaparib (25 mg/kg) i.p. daily for 2–4 weeks (3) Olaparib (25 mg/kg) i.p. every other day for 4 weeks	(1) - (2) - (3) 0.4% lithium carbonate-containing diet	(1) Olaparib increased survival in Kasumi model. (2, 3) Olaparib did not increase survival in THP-1 or MLL-driven AML PDX models. (3) Combination increased survival	(1) + (2, 3) -	++	[56]
	Female C57BL/6J mice, AML cells i.v.	Olaparib (50 mg/kg) orally for 5 days	AZD1775 (80 mg/kg) orally for 5 days	Olaparib alone slightly extended survival. Combination significantly improved survival and reduced tumour burden	+	++	[55]
	Female and male NSG mice, patient-derived M4-AML sample i.v.	Rucaparib (1.3 mg/kg) i.p. daily for 5 days	5-FU (150 mg/kg) i.p. on day 2	Rucaparib alone did not increase survival. Combination with 5-FU increased survival	-	++	[45]
	Female NSG mice, MOLM-14 or MV-4-11 i.v.	Talazoparib (0.1 mg/kg) orally 5 days per week	Azacytidine (0.5 mg/kg) s.c. 5 days per week	Combination decreased tumour burden and increased survival	-	++	[59]
	NSG mice, patient-derived AML cells i.v.	Talazoparib (0.33 mg/kg) orally for 7 days	Doxorubicin (1.5 mg/kg) i.v. on days 1–3 and cytarabine (50 mg/kg) i.v. on days 1–5	Talazoparib alone did not increase survival. Combination with doxorubicin and cytarabine increased survival	-	++	[63]
	NRGS mice, patient-derived AML-FLT3ITD sample i.v.	Talazoparib (0.33 mg/kg) daily for 7 days	AC220 (10 mg/kg) for days	Talazoparib alone had no effect. Combining talazoparib and AC220 significantly increased survival	-	++	[62]
	Female NOD.CB17-Prkdc^scid^/J mice, patient-derived FLT3-ITD samples i.v. or *Tet2*^−/−^ AML-like murine leukaemias i.v.	Talazoparib (0.165 mg/kg) i.v. for 7 days	Imatinib (100 mg/kg) daily for 7 days or Quizartinib (1 mg/kg) daily for 7 days	Combined PARPi and imatinib or Quizartinib increased survival	N/A	++	[64].
	NSGS mice, U937 wild-type and *STAG2*-knockout	Talazoparib (0.25 mg/kg) oral daily	-	Decreased leukaemic burden in *STAG2* knockout cells	+	N/A	[96]
	Female C57BL/6 mice, RFP-GFP-double positive leukaemic spleen cells from mice that underwent secondary/ tertiary transplantation that developed acute erythroid leukaemia	Talazoparib (0.1 mg/kg) oral days 1–5 and days 14–19	Decitabine (0.5 mg/kg) i.v. daily days 1–5	Combination with decitabine significantly decreased spleen size, but not survival	+	++	[97]
Chronic Leukaemias							
CML	NSG mice, patient-derived CML-CP or CML-AP samples i.v.	Talazoparib (0.33 mg/kg) orally for 14 days	Imatinib (100 mg/kg) orally twice daily	Talazoparib alone increased survival. Combination with imatinib significantly increased this effect	+	++	[63]
	NOD.*Rag1^−/−^*;γc*^null^* mice, patient-derived CML-CP samples i.v.	5F02 (2.5 mg/kg) i.p. and/or Talazoparib (0.33 mg/kg) i.v.	Imatinib (100 mg/kg) orally twice daily 7 days and	Combined administration of imatinib and PARPi reduced tumour burden	N/A	++	[68]
	Total body irradiated (600 cGy) mice, patient-derived CML-CP samples pre-treated with drugs i.v.	Pre-treatment with talazoparib (100 nM)	Pre-treatment with imatinib (1 µM)	Pre-treatment with the combination prevented engraftment	+	++	[70]
	Male BALB/c nude mice, patient-derived CML sample s.c.	Talazoparib (50 mg/kg) orally daily	Chloroquine (50 mg/kg) i.p. daily	Talazoparib decreased tumour burden. Combination with chloroquine synergistically decreased tumour volume	+	++	[71]
Lymphomas							
CLL	Conditional deletion of *ATM* in B-cells in *Eµ: TCL1* mice on a mixed C57BL/6J-C57BL/6N background	Olaparib (50 mg/kg) i.p. 5 days per week	-	Increased survival and improved spleen volume	+	N/A	[98]
Murine Lymphoma	Male C57BL/6 × DBA/2 mice, L5178Y cells intracranially	NU1025 (1 mg/mouse) intracranially	TMZ (100 mg/kg or 200 mg/kg) i.p.	NU1025 alone had no effect on survival. Combining with TMZ significantly increased survival	-	++	[99]
NHL	NOD/SCID mice, Granta-519 i.v.	Olaparib (50 mg/kg) i.p. for 14 days	-	Olaparib decreased tumour engraftment and increased survival	+	N/A	[73]
	Female RAG2^−/−^ mice, Granta-519 or Z138 s.c.	Olaparib (25 mg/kg or 50 mg/kg) i.p. for 28 days	-	Delayed tumour growth and increased survival	+	N/A	[76]
	Female RAG2^−/−^ mice, Granta-519 or UPN2 s.c.	Olaparib (50 mg/kg) i.p. for 28 days	-	Delayed tumour growth and increased survival	+	N/A	[77]
	(1) NOD-SCID mice, OCI-LY19, OCI-LY1, or OCI-LY8 cells s.c., (2) NSG mice, patient-derived samples s.c.	Olaparib (50 mg/kg) i.p. daily	R-CHOP: Rituximab (20 mg/kg) + cyclophosphamide (40 mg/kg) + Doxorubicin (3.3 mg/kg) + vincristine (0.5 mg/kg) i.v. on day 1, prednisone (0.2 mg/kg) orally for 5 days	Olaparib alone increased survival in all models. When combined with R-CHOP in OCI-LY8 xenografts delayed tumour growth and significantly improved survival	+	++	[80]
	Female NSG mice, patient-derived Burkitt lymphoma samples i.v.	Talazoparib (0.33 mg/kg) orally for 7 days	Cytarabine (50 mg/kg) i.v. on days 1–5	Talazoparib monotherapy decreased tumour burden and increased survival. Combining talazoparib and cytarabine synergistically enhanced effects	+	++	[78]
Cutaneous lymphoma	C57Bl/6 mice, MBL cells dermal injection	(1) Talazoparib (dose not reported) i.p. for 7 days (2) Talazoparib (dose not reported) orally for 7 days	(1) - (2) Romidepsin (dose not reported) i.p. for 7 days	Talazoparib treatment significantly reduced tumour volume. Combination with romidepsin significantly increased this effect	+	++	[85]
Multiple Myeloma							
	Male CB-17 SCID mice, H929 or bortezomib resistant AMO1 cells s.c.	Olaparib (100 mg/kg) orally daily	-	Reduced tumour growth in both xenograft models	+	N/A	[89]
	CB-17 SCID mice, MM.1S cells s.c.	Veliparib (50 mg/kg) orally twice daily	Bortezomib (0.4 mg/kg) s.c. twice per week	Combination decreased tumour burden and increased survival	-	++	[91]
	Male CB-17 SCID mice, MM.1S cells s.c.	Veliparib (50 mg/kg) orally twice daily, 5 days per week	Dinaciclib (35 mg/kg) i.p. twice per week	Veliparib alone had no effect on tumour volume or survival. Combining with dinaciclib delayed tumour growth and improved survival	-	++	[90]
Myeloproliferative Neoplasms							
Murine MPN-like disease	C57BL/6 mice injected with GFP+JAK2^V617F^ and WT bone marrow cells	Talazoparib (0.33 mg/kg) i.v. daily	Hydroxyurea (30 mg/kg) i.p. twice daily, and/or ruxolitinib (30 mg/kg) orally twice daily	Talazoparib decreased tumour burden. Combining with hydroxyurea and/or ruxolitinib significantly increased these effects	+	++	[94]
ET	NSG mice, SET2 cells i.v.	Veliparib (3 mg/kg) i.p. for 5 days	Busulfan (25 mg/kg) i.p. weekly	Veliparib alone had no effect on survival, but combining with busulfan significantly increased survival	-	++	[93]
MDS	Sequential bone marrow transplant to generate *Tet2/STAG2* mutant mice	Talazoparib (0.25 mg/kg) orally daily	-	Talazoparib selectively depleted cohesin-mutant cells	+	N/A	[96]

ALL, acute lymphoblastic leukaemia; AML, acute myeloid leukaemia; *ATM*, ataxia telangiectasia mutated; CLL, chronic lymphoblastic leukaemia; CML, chronic myeloid leukaemia; ET, essential thrombocythaemia; ENU, N-ethyl-N-nitrosourea; 5-FU, 5-fluorouracil; i.f., intrafemoral; i.p., intraperitoneal; i.v., intravenous; MM, multiple myeloma; MPN, myeloproliferative neoplasms; NSG, NOD scid gamma; PARPi, PARP inhibitor; s.c., subcutaneous; TMZ, temozolomide.

**Table 4:** Summary of clinical trials investigating PARPi in haematological malignancies.

Cancer Types	Clinical Trials Identifier	Phase	Treatment Regimen	Effects	Refs
Advanced solid tumours (*n* = 4), NHL (*n* = 3), cutaneous T-cell lymphoma (*n* = 2)	-	Phase 0	Veliparib (10, 25 or 50 mg) single oral dose	Veliparib treatment decreased PAR and the ratio of PAR to PARP-1 in tumour cells	[116]
Advanced solid tumours (*n* = 8), low grade lymphoma (*n* = 3), cutaneous T-cell lymphoma (*n* = 3)	NCT00387608	Phase 0	Veliparib (10, 25, 50, 100, or 150 mg) single oral dose	Good oral bioavailability and was well tolerated. Significant inhibition of PAR levels in tumour cells at the 25 and 50 mg doses	[117]
Colorectal cancer (*n* = 5), ovarian (*n* = 5), melanoma (*n* = 2), pancreas (*n* = 1), endometrial cancer (*n* = 1), Hurthle cell thyroid (*n* = 1), other (*n* = 8; pleural mesothelioma, hepatocellular, NHL, external ear adenocarcinoma, bile duct adenocarcinoma, small-cell lung cancer, oesophageal adenocarcinoma, chondrosarcoma)	NCT00553189	Phase I	Cohort 1: 10 mg po BID veliparib days 1–7 + 1.2 mg/m^2^/d i.v. topotecan days—8, 2–5 (cycle 1) and days 1–5 (cycle 2 onwards (*n* = 6); Cohort 2: 10 mg po BID veliparib days 1–7 + 0.9 mg/m^2^/d i.v. topotecan days—8, 2–5 (cycle 1) and days 1–5 (cycle 2 onwards (*n* = 3); Cohort 3: -2: 10 mg po BID veliparib days 2–5 (cycle 1) and days 1–5 (cycle 2 onwards) + 0.75 mg/m^2^/d i.v. topotecan days 1–5 (*n* = 3); Cohort 4: −3: 10 mg po BID veliparib days 2–5 (cycle 1) and days 1–5 (cycle 2 onwards) + 0.6 mg/m^2^/d i.v. topotecan days 1–5 (*n* = 4 + 3); Cohort 5: 10 mg po BID veliparib day 1 + 0.75 mg/m^2^/d i.v. topotecan days 1–5 (*n* = 5)	Most common DLTs: grade 4 neutropaenia and thrombocytopaenia, grade 4 neutropaenia lasting >5 days, febrile neutropaenia, grade 3 or 4 myelosuppression. Four of 6 patients (66.7%) on dose level 1 had stable disease after 2 cycles (taken off study due to toxicity)	[118]
Advanced solid tumours (*n* = 33) and refractory lymphoma (*n* = 2)	NCT00810966	Phase I	Cohort 1: 20 mg veliparib QD × 7 days + 50 mg cyclophosphamide QD × 21 days (*n* = 3); Cohort 2: 30 mg veliparib QD × 7 days + 50 mg cyclophosphamide QD × 21 days (*n* = 3); Cohort 3: 30 mg veliparib QD × 14 days + 50 mg cyclophosphamide QD × 21 days (*n* = 3); Cohort 4: 40 mg veliparib QD × 21 days + 50 mg cyclophosphamide QD × 21 days (*n* = 3); Cohort 5: 40 mg veliparib QD × 21 days + 100 mg cyclophosphamide QD × 21 days (*n* = 3); Cohort 6: 50 mg veliparib QD × 21 days + 50 mg cyclophosphamide QD × 21 days (*n* = 3); Cohort 7: 60 mg veliparib QD × 21 days + 50 mg cyclophosphamide QD × 21 days (*n* = 14); Cohort 8: 80 mg veliparib QD × 21 days + 50 mg cyclophosphamide QD × 21 days (*n* = 3)	Generally well tolerated. Grade 2 myelosuppression was the most common toxicity. Twelve out of 35 (34%) patients exhibited grade 3 or 4 lymphopaenia. Seven out of 35 (20%) of patients experienced a partial response, and 6/35 (17%) exhibited prolonged stable disease, including 1 patient with lymphoma	[119]
De novo or secondary AML (*n* = 48)	NCT01139970	Phase I	Veliparib 20–200 mg PO day 1 and BID days 5–12 (cycle 1) or days 1–8 (cycle 2 onwards) + 150–200 mg/m^2^/d PO days 3–8 (cycle 1) or days 1–5 (cycle 2 onwards) every 28–56 days	No DLT observed for 20–150 mg veliparib. A DLT of grade 3 oropharyngeal mucositis/esophagitis was observed in 2/4 (50%) of patients treated with 200 mg veliparib. The most common serious adverse events were infections (40%), febrile neutropaenia (25%), and oropharyngeal mucositis/esophagitis (4%). Complete responses were attained in 8/48 (16.6%) patients, with 7/8 achieving complete remission after a single cycle. An additional 8/48 (16.6%) patients exhibited disease stabilisation	[115]
Relapsed refractory AML, newly diagnosed aggressive MPN, aggressive CMMoL (*n* = 99)	NCT03289910	Phase I	Veliparib PO BID with dose escalation (10–100 mg × 8 days; 80 mg × 14 or 21 days) + 1–1.3 mg/m^2^/d topotecan continuous i.v. days 3–7 + 120–150 mg/m^2^/d carboplatin continuous i.v. days 3–7	Response rate in AML with no history of MPN or CMMoL was 25% (19/77). Response rate in aggressive MPN, CMMoL or related AML was 64% (14/22). Mucositis was dose limiting and correlated with high veliparib concentrations	[120]
Relapsed/refractory lymphoma (*n* = 23; cHL, DLBCL, FL, tFL), MM (*n* = 1), and advanced solid malignancies (*n* = 25)	NCT01326702	Phase I/II	Dose-escalation cohorts (*n* = 34): 200–400 mg PO BID veliparib days 1–7 of 28-day cycle + 70 and 90 m^2^/d bendamustine i.v. days 1 and 2; Cohort expansion (*n* = 7) in B-cell lymphomas: 300 mg PO BID veliparib + 90 mg/m^2^ bendamustine and 375 mg/m^2^ rituximab day 1	DLTs: anaemia, nausea, hypertensions, and hyperhidrosis. Most common grade 3–4 toxicity was lymphopaenia (88%), anaemia (20%), neutropaenia (12%), thrombocytopaenia (10%), and leucopoenia (10%). Five of seven (71%) lymphoma patients on veliparib + bendamustine and 6/7 (86%) on veliparib + bendamustine + rituximab achieved objective response. Patient with MM achieved a partial response	[121]
Relapsed CLL (*n* = 9), MCL (*n* = 4) and T-PLL (*n* = 2)	ISRCTN34386131	Phase I	Cohort 1: 200 mg capsule olaparib PO BID (*n* = 6); Cohort 2: 400 mg olaparib capsule PO BID (*n* = 3); Cohort 3: 100 mg olaparib tablet PO BID (*n* = 6)	Myelosuppression was the most common haematological grade 3–4 toxicity (*n* = 8). Overall, both formulations of olaparib were well tolerated, with the most common AEs being anaemia (66%), thrombocytopaenia (53%), fatigue (53%), nausea (33%) and neutropaenia (20%). All 3 patients who received the higher dose of 400 mg BID capsules developed DLTs possibly attributable to olaparib. The median OS for patients treated with capsules (106 days) was not dissimilar to that for patients treated with tablets (129 days)	[122]

AE, adverse events; cHL, classical Hodgkin Lymphoma; CLL, chronic lymphoblastic leukaemia; CMMoL, chronic myelomonocytic leukaemia; DLBCL, diffuse large B-cell lymphoma; DLT, dose-limiting toxicity; FL, follicular lymphoma; tFL, transformed follicular lymphoma; MCL, mantle-cell lymphoma; MM, multiple myeloma; MPN, myeloproliferative neoplasm; NHL, non-Hodgkin lymphoma; OS, overall survival; T-PLL, T-prolymphocytic leukaemia.
